# A comprehensive model combining radiomics and risk factors for predicting massive hemorrhage in cesarean scar pregnancy during dilatation and curettage

**DOI:** 10.1016/j.ejro.2025.100661

**Published:** 2025-06-04

**Authors:** Feng Gao, Le Fu, Zhuoying Zhang, Yafen Li, Zeyi Zhang, Yong Zhang, Yichen Zhang, Jie Shi, Jiejun Cheng

**Affiliations:** a Department of Radiology, Shanghai First Maternity and Infant Hospital, Tongji University School of Medicine, Shanghai, China; b School of Health Science and Engineering, University of Shanghai for Science and Technology, Shanghai, China; c MR Research, GE Healthcare, Shanghai, China

**Keywords:** Caesarean scar pregnancy, Magnetic resonance imaging, Radiomics, Dilation and curettage, Massive hemorrhage, Nomogram

## Abstract

**Background:**

To develop a comprehensive model integrating MRI radiomics signatures and independent risk factors for predicting the risk of massive bleeding during dilatation and curettage(D&C) in patients with cesarean scar pregnancy (CSP).

**Methods:**

CSP patients who underwent D&C were retrospectively reviewed. Intraoperative massive bleeding was defined as bleeding exceeding 200 ml based on surgical records. Three-dimensional MRI T2-weighted images were obtained, and radiomics signatures were extracted from the gestational sac (GS). Subjects were randomly separated into the training and testing sets in a 7:3 ratio. Radiomics features and clinical variables were analyzed to conduct both radiomics and clinical models. The nomogram was established by combining Radscore and the selected clinical variables.

**Results:**

Among 109 CSP patients, 33 patients experienced massive hemorrhage while 76 patients did not. Serum β-hCG and the maximum inlet diameter of the CSD (P < 0.05) were identified as significant clinical prognostic factors for massive hemorrhage. The nomogram demonstrated superior AUCs of 0.962 (95 % CI 0.928–0.989) and 0.926 (95 % CI 0.843–0.987) in the training and testing cohorts, respectively, Delong’s test was employed to compare the AUCs of the nomogram with those of the radiomics model and the clinical model. The results showed no significant differences between the nomogram and the other models in both the training (p > 0.05) and testing cohorts (p > 0.05). The nomogram calibration curve exhibited good agreement, with no significant differences found in the Hosmer-Lemeshow test (all p > 0.05). DCA revealed a substantial overall net benefit for the nomogram.

**Conclusions:**

Our study achieved accurate prediction of massive hemorrhage during D&C in CSP patients by integrating MRI radiomics and clinical features, underscoring the synergistic effectiveness of radiomics combined with clinical variables. The combined nomogram offered valuable support for precise preoperative risk assessment and individualized treatment decisions.

## 1 Background

Cesarean scar pregnancy (CSP) is a unique type of ectopic pregnancy wherein the gestational sac (GS) implants on the scar tissue from a previous cesarean section incision[1]. The incidence of CSP has been increasing, which correlates with the rising rate of cesarean sections[2]. Managing CSP remains challenging due to the complexity of clinical conditions and the variability in CSP types, resulting in a lack of consensus on the optimal treatment approach[3], [4], [5]. Treatment options for CSP include dilation and curettage (D&C), local resection of the pregnancy through laparotomy, laparoscopy, and/or hysteroscopy, uterine artery embolization (UAE), and hysterectomy. Among these, D&C is considered one of the most effective and convenient treatment options for CSP[4], and it is the primary method used in our institution. However, it is important to note that these procedures carry potential risks, such as intraoperative blood loss, retained products of conception, or postpartum hemorrhage[2], [6], [7], [8]. These complications can lead to increased patient trauma, impaired fertility, and even life-threatening conditions. Therefore, identifying individuals at high risk for intraoperative bleeding before undergoing D&C is crucial. Implementing screening protocols can aid in treatment decision-making and enhance doctor-patient communication, ultimately aiming to reduce complications.

Ultrasound is commonly utilized as the primary imaging modality for the initial diagnosis and evaluation of CSP. However, the accuracy of ultrasound findings can vary depending on the expertise and experience of the examiners[6], [7], [8]. In recent years, the significance of MRI in assessing CSP patients has been increasingly acknowledged[9], [10], [11]. MRI offers multi-directional imaging capabilities and superior soft tissue resolution, allowing for clear visualization of the relationship between the GS and the surrounding tissues[12], [13]. However, direct observation is susceptible to observer influence, impacting the precision and reliability of massive bleeding predictions. Subsequent studies have shown that detailed features derived from the MRI, such as the thickness of the cesarean scar (CS), the diameter of GS, and the area of cesarean section diverticulum (CSD), are associated with intraoperative massive bleeding [14], [15]. Despite this, the measurer can easily influence these factors, limiting the accuracy of predictions.

Radiomics can extract high-throughput statistics from medical images and combine them with patient information. Utilizing advanced bioinformatics tools, these data can be used to develop models that enhance diagnostic, prognostic, and predictive accuracy by uncovering hidden relationships between image features and underlying pathophysiology[16]. Radiomics has been wildly used in the diagnosis and prognosis prediction of malignant tumors, aiding in distinguishing patients and predicting outcomes [17], [18], [19], [20]. To the best of our knowledge, no published manuscript to date has examined the application of radiomics in predicting massive bleeding during D&C for CSP patients.

Therefore, the objective of this study was to develop a radiomics nomogram model that can accurately predict the occurrence of massive hemorrhage before D&C in patients with CSP, to ultimately improve preoperative risk assessment and enhance patient management for CSP.

## 2 Materials and methods

### 2.1 Study population

This retrospective study received approval from the Ethics Committee of our hospital (registration number: KS22281), and patient informed consent for data use and publishing was waived because of the retrospective nature of our study. Written informed consent for pelvic MRI scans was obtained from all patients before the MRI examination.

**Fig. 1:**
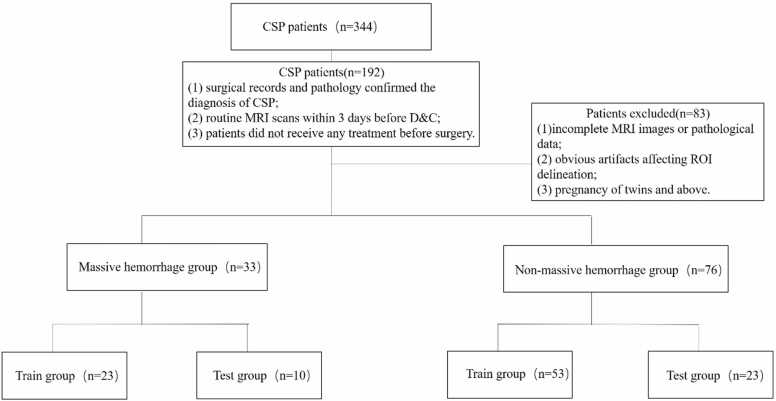
The flowchart of participant selection.

Patients were included based on the following criteria: (1) surgical records and pathology confirmed the diagnosis of CSP; (2) routine MRI scans within 3 days before D&C; (3) patients did not receive any treatment before surgery. The following exclusion criteria were applied: (1) incomplete MRI images or pathological data; (2) obvious artifacts that affected the delineation of the regions of interest (ROI); (3) pregnancies involving twins or higher-order multiples. Finally, a total of 109 patients from January 2019 to December 2022 were enrolled. The patient selection process is outlined in Fig. 1.

These patients were categorized into two groups based on the amount of intraoperative blood loss. The Massive Hemorrhage (MH) group was defined as having an intraoperative blood loss of ≥ 200 ml, while the remaining patients (with an intraoperative blood loss of < 200 ml) were classified as the Non-Massive Hemorrhage (Non-MH) group [22].

## 3 MRI examination

All MRI examinations were performed on a 1.5 T system (OPTIMA MR360, GE Healthcare, Milwaukee, WI) in the supine position using an 8-channel phased-array coil. No gadolinium was administered in any case. The sagittal T2W CUBE sequence was selected for this study due to its clear visualization of the gestational sac (GS) and adjacent tissues, especially the relationship between the GS and the cesarean scar defect (CSD). This imaging sequence provides superior soft tissue contrast, facilitating accurate region-of-interest (ROI) delineation and radiomics feature extraction. Additionally, the sagittal orientation enables thorough evaluation of uterine anatomy along its longitudinal axis, aiding in the identification of abnormalities associated with cesarean scar pregnancy (CSP). The acquisition parameters were as follows: repetition time/echo time [TR/TE], 2000/91–95minimum msec; slice thickness, 1.6 mm; intersection gap:0; matrix size, 228 × 228; field of view: 240 × 240.

## 4 Clinical features

**Fig. 2:**
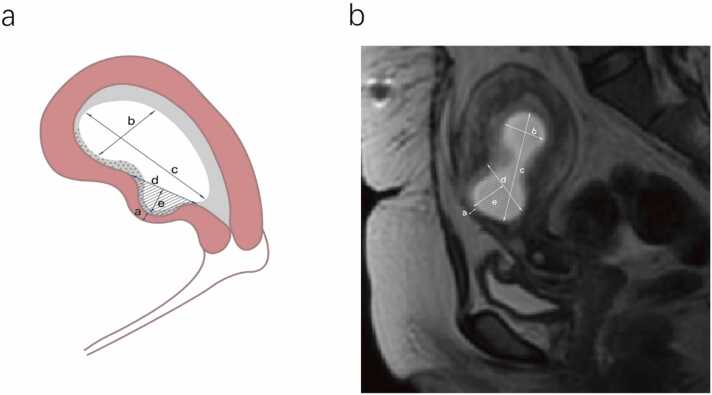
Illustration of the measurements for the dimensions of the CSD and GS. a: the minimum wall thickness of the CSD defect; b: the maximum transverse diameter of the GS; c: the maximum longitudinal diameter of the GS; d: the maximum inlet.

The baseline clinical data were retrieved from medical records, including patient age, pregnancy history, fertility history, number of CS, gestational age, and preoperative serum β human chorionic gonadotropin (β-hCG) levels. Two experienced radiologists, each having a minimum of five years in gynecology MRI, utilized 3D Slicer (v5.6.1, https://download.slicer.org/) to measure the following MR findings on sagittal MR images: the minimum wall thickness of the CSD defect(a), the maximum transverse diameter of the GS(b), the maximum longitudinal diameter of the GS(c), the maximum inlet diameter of the CSD(d), the maximum depth of the CSD(e), and area of the CSD (Fig. 2). These measurements and clinical data were gathered for further analysis and modeling.

### 4.1 Image analysis and radiomics feature extraction

**Fig. 3:**
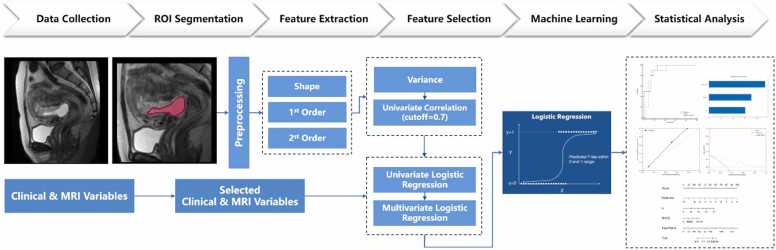
Radiomics workflow of this study.

Fig. 3 shows the workflow of the GS radiomics nomogram for CSP patients. The volumetric region of interest of the GS (VOI, delineating along the GS margin) was manually segmented on the sagittal T2W CUBE images using 3D Slicer(v5.6.1, https://download.slicer.org/). To preprocess the images and extract radiomics features, we employed PyRadiomics (v 3.0.1, https://pyradiomics.readthedocs.io). The images were normalized using the z-score normalization method, based on the signal intensity of each MRI image. Gray-level discretization was performed using fixed bin width values of 25. To achieve a standardized spatial resolution, the voxel size was resampled to 1 mm × 1 mm × 1 mm. Seven classes of radiomics features were automatically extracted from original images and filtered images (Laplacian of Gaussian filter, wavelet filtered, and 3D local binary pattern filtered). Consequently, 1502 three-dimensional radiomics features were obtained.

### 4.2 Feature dimension reduction and radiomics signature construction

All subjects were then randomly divided into a training set (N = 76) and a test set (N = 33) at a ratio of 7:3. Feature selection, radiomics signature construction, and machine learning model construction were performed on the training set and tested on the testing set. Before conducting analyses, the radiomics features were normalized using the z-score method. To minimize the chance of overfitting, we reduced the dimension of radiomics features by a sequential process, including: (1) retaining variables with non-zero variance for further analysis; (2) conducting univariate Spearman correlation analysis between each feature, and removing features with a correlation higher than 0.7 to reduce multicollinearity; (3) performing general univariate analysis with a significance threshold of 0.05, features with significant difference (p < 0.05) remained. The choice between Mann-Whitney *U* test or analysis of variance (ANOVA) was made automatically depending on the normality and homogeneity of variance of the variables; (4) employing multivariate logistic regression analysis to select the most useful predictive features. A radiomics signature (Radscore) was computed for each patient by combining the remaining radiomics features with their respective weight coefficients calculated from multivariate logistic regression analysis. For clinical features, the independent risk factors were identified using univariate and multivariate logistic regression analyses.

#### 4.2.1 Machine learning model construction

The input of the Radiomics model (Model 1) and clinical model (Model 2) were Radscore and independent risk factors of clinical features, respectively. The combined nomogram model (Model 3) was constructed by combining significant independent risk factors and the Radscore. Logistic regression was applied to three models in the training cohort, using 5-fold cross-validation to select the optimal model parameters. Then the models were subsequently validated in the test cohort.

### 4.3 Statistical analysis

All statistical analyses were performed using SPSS (version 26.0) and Python (version 3.8.8). The difference tests for continuous clinical features were determined based on the normality and homogeneity of variance of their distributions: either an independent-sample *t*-test or Mann-Whitney *U* test was employed. The differences for discrete variables were compared by Chi-squared test. The receiver operating characteristic (ROC) curve and area under the curve (AUC) were acquired to evaluate the discrimination performance of the three models. Delong’s test was employed to compare the AUCs of different models. Accuracy, sensitivity, and specificity were calculated based on Youden’s J index. A calibration curve with the Hosmer—Lemeshow test was plotted to illustrate the goodness of fit for the model exhibiting the highest prediction performance on the test set. The clinical utility of this model was evaluated using decision curve analysis, helping assess the net benefit of using the model in clinical decision-making, details are presented in supplementary S1. A two-tailed p-value < 0.05 indicated statistical significance.The METRICS quality score of this study was calculated[23].

## 5 Results

### 5.1 Clinical characteristics

Table 1 indicates the clinical characteristics of CSP patients in this study. Thirty-three (mean age: 34.12 ± 5.18) of 109 patients were confirmed to be MH, while 76 (mean age: 34.83 ± 4.22) were non-MH. Eight clinical characteristics were found to have significant associations with the occurrence of MH during the intraoperative period, including gestational age, serum β-hCG, number of parity, and five MR measurements (b, c, d, e, and area of the CSD) (all p < 0.05). Univariate logistic analysis was conducted on both clinical and MR measurements in the training cohort, as presented in Table 2. Seven variables significantly associated with MH (all p < 0.001). The subsequent multivariate logistic analysis revealed that serum β-hCG levels and the maximum inlet diameter of the CSD were identified as independent risk factors for MH (both p < 0.01; Table 2).

**Table 1:** Demographic and clinical information of study subjects.

Characteristics	MH group(n = 33)	Non-MH group(n = 76)	p-value
Age (years)	34.12 ± 5.18	34.83 ± 4.22	0.455
Gestational age (days)	55.52 ± 11.84	45.32 ± 8.72	0.000
Serum *β*-hCG (mIU/ml)	123,018 ± 71,568.30	36,590 ± 41,361.01	0.000
Number of Gravidity	3.12 ± 1.64	3.20 ± 1.49	0.812
Number of parity	1.48 ± 0.62	1.31 ± 0.52	0.145
Number of cesarean sections	1.48 ± 0.57	1.25 ± 0.47	0.026
Maximum longitudinal diameter of the GS(mm)	35.04 ± 18.94	17.22 ± 10.54	0.000
Maximum transverse diameter of the GS(mm)	22.98 ± 13.92	10.96 ± 7.67	0.000
Minimum wall thickness of the CSD defect(mm)	3.48 ± 2.77	2.61 ± 2.86	0.143
Maximum inlet diameter of the CSD(mm)	36.42 ± 14.28	18.08 ± 9.21	0.000
Maximum depth of the CSD(mm)	19.90 ± 7.60	10.09 ± 6.30	0.000
Area of the CSD(mm^2^)	15.14 ± 26.80	1.61 ± 1.67	0.000

MH: massive hemorrhage, *β*-hCG: β human chorionic gonadotropin; GS: gestational sac; CSD: cesarean section diverticulum

**Table 2:** The univariate and multivariate logistic regression analyses for independent risk factors of massive hemorrhage in CSP patients.

Variables	Univariate analysis			Multivariate analysis	
	Odds ratio	95 %CI	p value	Odds ratio	95 %CI
Gestational age	2.915	(1.582, 5.370)	p < 0.001		
Serum *β*-hCG	5.385	(2.601, 11.145)	p < 0.001	3.126	(1.388, 7.042)
Number of Gravidity	0.886	(0.537, 1.462)	0.635		
Maximum longitudinal diameter of the GS (c)	4.117	(1.947, 8.705)	p < 0.001		
Maximum transverse diameter of the GS (d)	4.793	(2.191, 10.486)	p < 0.001		
Maximum inlet diameter of the CSD (b)	8.144	(3.088, 21.474)	p < 0.001	4.639	(1.697, 12.682)
Maximum depth of the CSD (e)	4.097	(1.995, 8.416)	p < 0.001		
Area of the CSD	448,986.384	(435.395, 463,002,165.466)	p < 0.001		

*β*-hCG: β human chorionic gonadotropin; GS: gestational sac; CSD: cesarean section diverticulum; CI: confidence interval

## 6 Radscore building

Five radiomics features were finally selected from the sagittal MR images, including one shape feature extracted from the original images and four second-order features from the wavelet images. The Radscore was derived through the summation of five selected features, each weighted by its respective coefficient. The formula of the Radscore and the details of the five radiomics features can be found in the supplementary S1. Bar diagrams in supplementary S2 visually represent the individual Radscores for each patient.

## 7 Prediction model construction

**Fig. 4:**
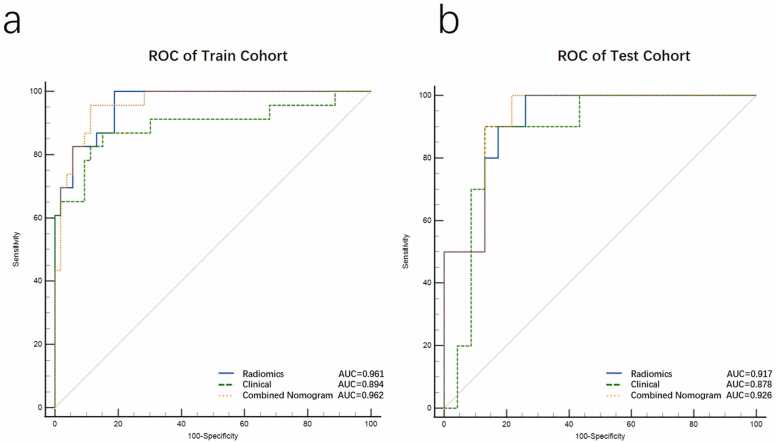
The diagnostic performance of the nomogram, radiomics, and clinical model for predicting massive hemorrhage in patients with CSP. The nomogram tended to yield the highest AUC in the train (a) and test (b) cohorts.

**Fig. 5:**
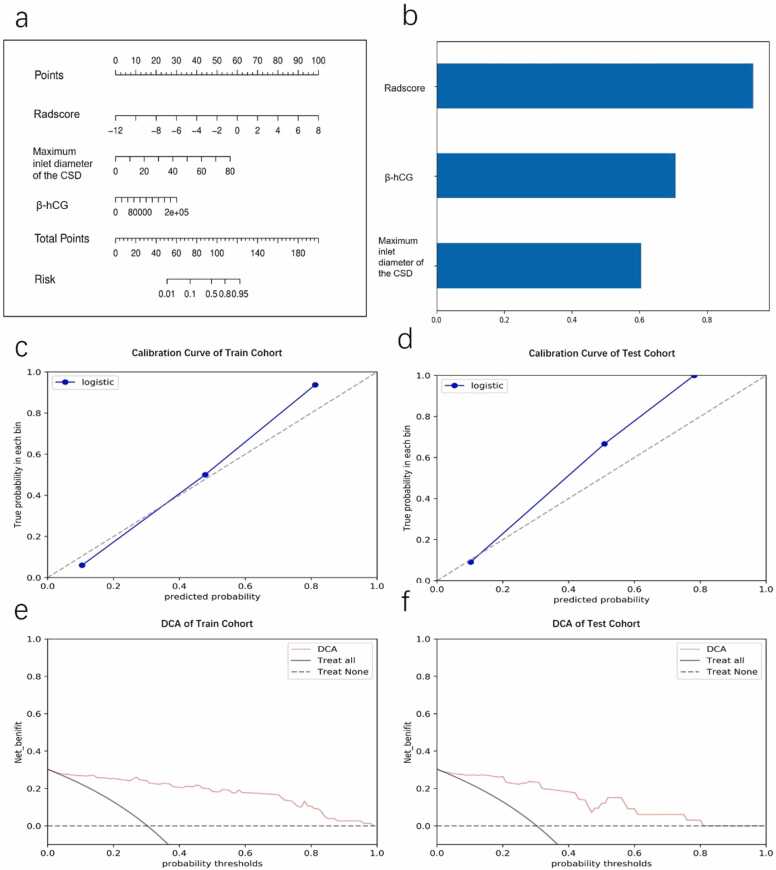
a) The nomogram constructed by integrating Radscore, β-hCG, and the maximum inlet diameter of the CSD. b) The feature importance of three features constructed the combined nomogram model.c, d) The calibration curves of the nomogram demonstrated good agreement in the train(c) and test(d) cohorts.e, f) The DCA curve of the nomogram in the train(e) and test(f) cohort.

Fig. 4 shows ROC curves comparing the radiomics model, clinical model and the combined nomogram. The radiomics model achieved AUC values of 0.961 (95 % CI: 0.925–0.987) in the training cohort and 0.917 (95 % CI: 0.829–0.981) in the testing cohort. The clinical model produced slightly lower AUC values of 0.894 (95 % CI: 0.81–0.969) in the training cohort and 0.878 (95 % CI: 0.762–0.975) in the testing cohort. Comparatively, the combined nomogram, constructed based on Radscore, serum β-hCG levels, and the maximum inlet diameter of the CSD (Fig. 5a,b), consistently outperformed both models. It exhibited the highest AUCs of 0.962 (95 % CI: 0.928–0.989) and 0.926 (95 % CI: 0.843–0.987) in the training and testing cohorts, respectively (Table 3). Delong’s test revealed no significant differences (supplementary S3).

**Table 3:** The diagnostic performance of combined nomogram, Radscore, and clinical model for predicting massive hemorrhage.

Model	Cohort	AUC	Accuracy	SEN	SPE	PPV	NPV	F1score
Radscore model	Train	0.961（0.925–0.987）	0.855	0.957	0.811	0.688	0.977	0.800
	Test	0.917（0.829–0.981)	0.848	0.800	0.870	0.727	0.909	0.762
Clinical model	Train	0.894(0.81–0.969)	0.842	0.826	0.849	0.704	0.918	0.760
	Test	0.878(0.762–0.975)	0.818	0.900	0.783	0.643	0.947	0.750
Combined nomogram	Train	0.962(0.928–0.989)	0.895	0.913	0.887	0.778	0.959	0.840
	Test	0.926(0.843–0.987)	0.848	0.900	0.826	0.692	0.950	0.783

AUC: area under the curve; SEN: sensitivity; SPE: specificity; PPV: positive predictive value, NPV: negative predictive value

Consent for publication

Not applicable

**Fig. 6:**
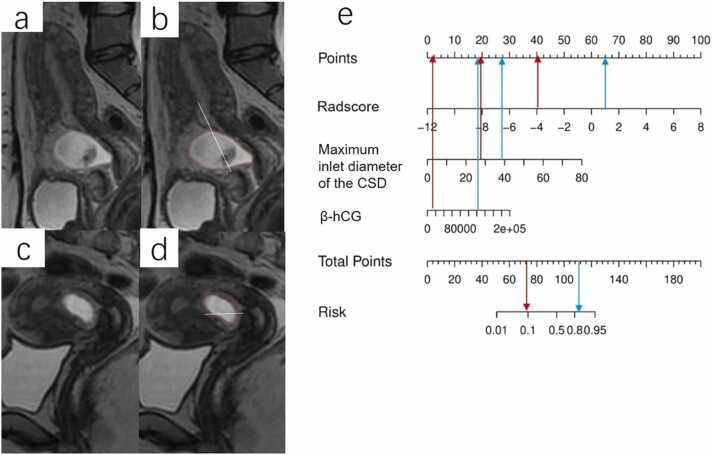
Image examples of CSP patients with massive hemorrhage and minor hemorrhage, and application of nomogram to predict the probability of massive hemorrhage. The nomogram shows points assigned for each predictor. The total number of points is calculated by adding points assigned for all variables and is then used to determine the corresponding risks of massive hemorrhage. a, b) A massive hemorrhage CSP patient’s MR shows the ROI of the GS (red circle) and the maximum inlet diameter of the CSD(white line). c, d) A minor hemorrhage CSP patient’s MR shows the ROI of the GS(red circle) and the maximum inlet diameter of the CSD(white line). e) Nomogram shows the determination of the risk of massive hemorrhage in both patients. For patient one (Fig. a and b, blue arrows), the nomogram yields a total of 111.5 points and a corresponding risk of massive hemorrhage of greater than 0.83. For patient two (Fig. c and d, red arrows), the nomogram yields a total of 72.5 points and a corresponding risk of massive hemorrhage of less than 0.1. Thus, the nomogram rendered correct predictions in both patients.

The METRICS quality score of this study is 6.8. The calibration curve of the nomogram and the nonsignificant results of the H-L test (both p > 0.05), revealed good agreement between predicted and observed outcomes in both the train and test cohorts (Fig. 5c,d). Fig. 5e,f demonstrates the DCA of the nomogram, indicating its potential clinical utility across a threshold probability range of 0.05–0.80. For example, at a threshold probability of 0.05, the model identifies individuals with a relatively low likelihood of massive hemorrhage, prompting clinicians to consider additional monitoring or diagnostic tests for further assessment. Conversely, at a threshold probability of 0.80, the model can flag individuals with a high predicted risk, indicating the need for more aggressive interventions or treatments. As revealed by DCA, this nuanced insight into the model's performance at varying probability thresholds empowers clinicians to tailor decision-making strategies based on the specific clinical context and desired levels of certainty. Fig. 6 depicts MRI images of representative patients with and without massive hemorrhage, presenting interpretations by two radiologists and corresponding nomogram results.

## 8 Discussion

In this study, we developed and evaluated three prediction models: the radiomics model, the clinical model, and the combined nomogram model, to assess the risk of preoperative massive hemorrhage in patients with CSP. Our findings suggest that the combined model may have an advantage over the other two models, indicating that integrating radiomics with clinical characteristics could improve the prediction of massive hemorrhage risk in CSP patients. The combined nomogram model, which incorporates the radscore, serum β-hCG level, and maximum inlet diameter of the CSD, appeared to be more effective in predicting massive hemorrhage risk, providing a user-friendly and personalized prediction tool.

The serum β-hCG level was identified as an independent risk factor for massive hemorrhage in our study, consistent with previous studies[21], [22]. In early pregnancy, rising serum β-hCG levels support the progression of pregnancy by stimulating the corpus luteum to produce increased progesterone. This ensures the optimal condition of the uterine lining for the nourishment and support of the developing embryo[24]. Moreover, β-hCG promotes the growth and invasion of trophoblastic cells, facilitating their invasion into the myometrium. Therefore, a higher serum β-hCG level indicates increased embryonic activity, implying a higher risk of intraoperative bleeding.

The significance of MRI measurements in evaluating and predicting intraoperative hemorrhage in CSP patients has garnered increasing attention in recent years[11], [15], [25], [26]. In our study, the maximum inlet diameter of the CSD proved to be a valuable predictor of massive hemorrhage. Consistent with previous research, this measurement is notably associated with type III CSP, which has a greater tendency for bleeding during surgery[13]. According to Chen’s study[27], when trophoblast cells are shallowly infiltrated into the myometrium, D&C may be considered safe and effective. We hypothesized that if the embryo was embedded deeper in the scar, the inlet diameter of the CSD would be enlarged by the GS and decidual layer. Consequently, the removal of the embryo could become challenging, leading to the potential for massive bleeding.

The radscore model yielded a high AUC and demonstrated good fitness in this study. Lai et al. identified three independent risk factors (uterine scar thickness, gestational sac diameter, and CSD area) and established an MRI-based scoring model for predicting intraoperative hemorrhage during D&C[11]. Despite its high predictive performance, their model exhibited underfitting, and the interobserver reliability for MRI features displayed considerable variability during pregnancy[28]. Therefore, a quantitative assessment of images is necessary for an objective evaluation. In our study, five radiomic features strongly correlated with massive bleeding were incorporated into the radscore model. While it may be challenging to interpret the specific underlying meaning of these radiomic features[29], the robust performance of the model in both the training and validation cohorts suggested that radiomics analysis served as a useful and stable tool for massive hemorrhage prediction.

The nomogram effectively stratified CSP patients according to their risk of massive bleeding during D&C. To the best of our knowledge, this is the first study to present a practical and convenient nomogram for preoperatively detecting massive bleeding in CSP patients. The readily available variables we utilized could serve as a convenient marker for predicting massive hemorrhage in CSP patients. Our intention was to assess the safety of D&C using this nomogram before surgery. In cases where we anticipated a high bleeding risk, we were inclined to administer preoperative treatments such as uterine artery embolization (UAE) before D&C to mitigate the risk of heavy bleeding[30]. Our goal was to provide an appropriate therapeutic approach to those patients with minimal trauma and cost.

Our results showed that a combined model integrating radiomics and clinical features can effectively predict the risk of massive hemorrhage in patients with cesarean scar pregnancy (CSP) undergoing dilatation and curettage (D&C). The radiomics approach extracts quantitative features from MRI images, providing clinicians with a more objective assessment tool that could enhance diagnostic accuracy and reliability. In clinical obstetric and gynecological practice, this model may assist physicians in identifying CSP patients at higher risk of severe bleeding during D&C, thus supporting preoperative risk evaluation and personalized treatment planning. For instance, if the model indicates a high risk of hemorrhage, physicians may consider preventive measures such as uterine artery embolization (UAE) prior to surgery. However, further research and validation are needed to confirm the feasibility and effectiveness of integrating this method into routine clinical workflows.

Our study had several limitations. First, the ROI only delineated the entire GS, and the exclusion of decidua may potentially impact model performance. Additionally, the risk of massive hemorrhage may vary with different types of CSP. However, our nomogram was developed using retrospective datasets, and the initial classification of CSP types was not available. Furthermore, the dataset of our study was relatively small and this was a single-center study. Future investigations should consider analyzing the decidua, incorporating CSP types, and utilizing larger datasets prospectively for a more comprehensive evaluation. In this study, the ROI only delineated the entire gestational sac, excluding the decidua, which may potentially impact the model performance. The decidua plays an important role in the pregnancy process as it is closely related to the development of the gestational sac and changes in the uterus. Existing studies have shown that changes in the morphology, structure, and function of the decidua may be associated with the occurrence, development of CSP, and the risk of bleeding during surgery[31]. Therefore, in future studies, considering the analysis of decidual characteristics and including them in the model may further improve the predictive performance and clinical application value of the model.

## 9 Conclusions

In conclusion, we developed a radiomics nomogram based on MRI phenotypes for predicting massive bleeding in CSP patients. The proposed nomogram holds valuable potential for identifying CSP patients at high risk of intraoperative massive hemorrhage during D&C, thereby facilitating the provision of additional perioperative care and preoperative preparation as needed.

## Ethics

This study was approved by the Ethics Committee of XXX. Written patient informed consent for the data used in our study was waived because of the retrospective nature of our study. The study was performed following the 1964 Helsinki Declaration and its later amendments or comparable ethical standards. The study complies with all regulations. All methods were performed under the relevant guidelines and regulations. Written informed consent for pelvic MRI scans was obtained from all patients before the MRI examination.

## Funding

This study was supported by grants from the Shang First Maternity and Infant Hospital Talent Project (2022RC04) and the Shanghai Tansuozhe Project (23TS1401000).

## CRediT authorship contribution statement

**Feng Gao:** Writing – review & editing. **Yichen Zhang:** Formal analysis. **Jie Shi:** Supervision, Software. **Yong Zhang:** Data curation. **Zeyi Zhang:** Investigation. **Yafen Li:** Software. **Zhuoying Zhang:** Methodology. **Le Fu:** Writing – original draft. **Jiejun Cheng:** Supervision.

## Declaration of Competing Interest

The authors declare the following financial interests/personal relationships which may be considered as potential competing interests: Jiejun cheng reports article publishing charges was provided by Shanghai first maternity and infant hospital. Reports a relationship with that includes. Has patent pending to. If there are other authors, they declare that they have no known competing financial interests or personal relationships that could have appeared to influence the work reported in this paper.

## Data Availability

The datasets generated and/or analyzed during the current study are not publicly available because they contain the patients’ personal information, but are available from the corresponding author on reasonable request.
