# Supplementary material for: A comprehensive model combining radiomics and risk factors for predicting massive hemorrhage in cesarean scar pregnancy during dilatation and curettage
Source: Eur J Radiol Open. 2025 Jun 4;14:100661. doi: 10.1016/j.ejro.2025.100661 (PMC12169770; doi:10.1016/j.ejro.2025.100661)
Supplement: Supplementary file 1 — Supplementary material [file mmc1.docx]

## S1

- Selected features:

1. wavelet-HHL_glszm_GrayLevelNonUniformity: a texture feature that quantifies the non-uniformity of gray levels in adjacent regions of an image after applying Haar wavelet transform and computing the Gray Level Size Zone Matrix (GLSZM). Higher values indicate greater variability in gray levels within the image region.
2. original_shape_Sphericity: a shape-based feature that characterizes the roundness of a three-dimensional object in a medical image. It is computed as the ratio of the surface area of a sphere with the same volume as the object to the surface area of the object.
3. wavelet-LLL_glcm_SumEntropy: a texture feature that is computed using the Gray-Level Co-occurrence Matrix (GLCM) applied to an image after applying a wavelet transform in the LLL (Low-Low-Low) subband. Specifically, it measures the sum of entropy in the GLCM, providing information about the randomness and disorder of pixel intensities in the specified wavelet subband. Higher values indicate greater complexity and variability in pixel relationships within that subband.
4. wavelet-HHL_gldm_LargeDependenceHighGrayLevelEmphasis: a texture feature that is computed by applying a wavelet transform to an image and then calculating the Large Dependence High Gray Level Emphasis (LDHGLE) from the Gray Level Dependence Matrix (GLDM) in the HHL (High-High-Low) subband. This feature provides information about the distribution of high gray-level values for large connected regions within the specified wavelet subband.
5. wavelet-HHL_glszm_SmallAreaLowGrayLevelEmphasis: is a texture feature that is computed by applying a wavelet transform to an image and then calculating the Small Area Low Gray Level Emphasis (SALGLE) from the Gray Level Size Zone Matrix (GLSZM) in the HHL (High-High-Low) subband. This feature provides information about the distribution of small connected regions with low gray-level values within the specified wavelet subband.

- Radscore

Radscore = -2.88684761 + 0.71693273*wavelet-HHL_glszm_GrayLevelNonUniformity – 2.47286799*original_shape_Sphericity – 2.43673115*wavelet-LLL_glcm_SumEntropy + 2.24211699*wavelet-HHL_gldm_LargeDependenceHighGrayLevelEmphasis + 1.91438822*wavelet-HHL_glszm_SmallAreaLowGrayLevelEmphasis

- Decision curve analysis (DCA)

The curve drawn with the threshold$Pt$as the x-axis and net profit as the y-axis is the DCA decision curve.

Net Benefit (NB) is defined as $A\times P－B\times L$, where A is the proportion of true positives, P is the benefit value of applying interventions to true positive patients, B is the proportion of false positives, and L is the loss value of applying interventions to false positive patients. The proportions here are calculated using all samples.

$Pt$is defined by $L/(P+L)$. It can be understood as the critical value used by the model to determine whether a patient is diseased. If the value is greater than the threshold, the patient is considered diseased; if it is less than the threshold, the patient is considered not diseased.

The dashed line (treat none) in the figure represents the scenario where all samples are negative and no one receives any intervention. In this case, regardless of the threshold, the net benefit of the intervention is zero. The solid black line (treat all) represents the scenario where all samples are positive, and everyone receives the intervention. This line shows the net benefit for this cohort under the model at different thresholds. is a method used to evaluate the clinical utility of predictive models. The area above the gray dashed line (treat none) and under the red curve indicates that the model has a positive net benefit at these thresholds. In other words, using this model to decide whether to proceed with treatment provides a greater benefit than not treating at all. From the figure, it can be seen that within a wide range of threshold values (Pt), the benefit of the model is higher than that of the extreme lines. Therefore, the range of selectable thresholds (Pt) for the model is relatively large, making it relatively safe. The appropriate threshold can be chosen based on the actual net benefit.

References:

1. Vickers, Andrew J, and Elena B Elkin. “Decision curve analysis: a novel method for evaluating prediction models.” Medical decision making : an international journal of the Society for Medical Decision Making vol. 26,6 (2006): 565-74. doi:10.1177/0272989X06295361
2. Vickers, Andrew J et al. “A simple, step-by-step guide to interpreting decision curve analysis.” Diagnostic and prognostic research vol. 3 18. 4 Oct. 2019, doi:10.1186/s41512-019-0064-7
3. Van Calster, Ben et al. “Reporting and Interpreting Decision Curve Analysis: A Guide for Investigators.” European urology vol. 74,6 (2018): 796-804. doi:10.1016/j.eururo.2018.08.038

## S2:


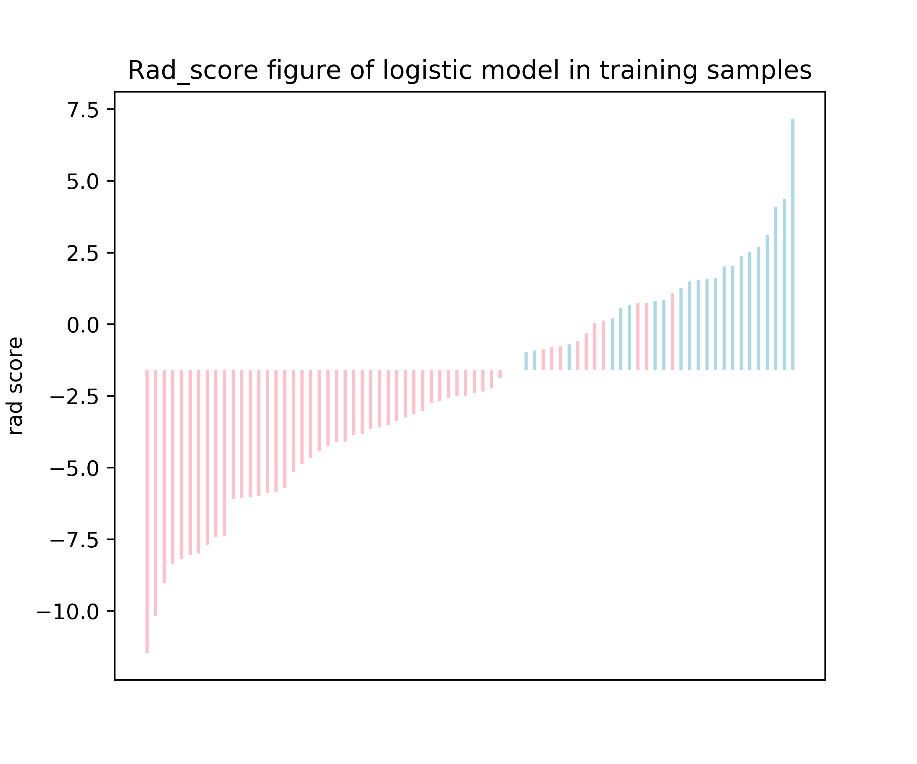


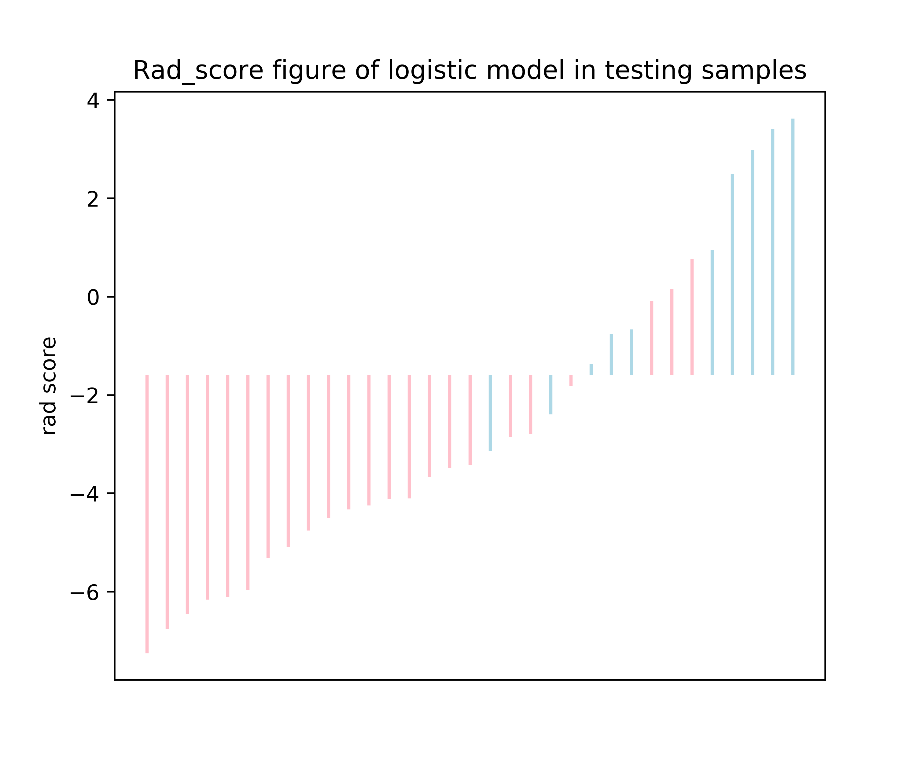


## S3

The significance level of pairwise comparison of ROC curves

|  | Train | Test |
| --- | --- | --- |
| Model 1 Vs Model 2 | 0.2292 | 0.6552 |
| Model 1 Vs Model 3 | 0.9542 | 0.5854 |
| Model 2 Vs Model 3 | 0.2234 | 0.8984 |
